# Case Report: Massive rectal bleeding from stercoral ulcers in a pediatric patient

**DOI:** 10.3389/fgstr.2026.1614154

**Published:** 2026-04-30

**Authors:** Paolo Quitadamo, Antonia Pascarella, Linda di Napoli, Maria Giovanna Puoti, Sara Isoldi, Ludovica Carangelo, Paolo Siani

**Affiliations:** 1 Pediatric Gastroenterology and Hepatology Unit, Santobono-Pausilipon Children’s Hospital, Naples, Italy; 2 Pediatric Department, Santobono-Pausilipon Children’s Hospital, Naples, Italy; 3 Department of Translational Medical Science, Section of Pediatrics, University “Federico II”, Naples, Italy; 4 Clinical Pharmacology and Toxicology Unit, Department of Neuroscience and Reproductive and Odonto-stomatological Sciences, University of Naples Federico II, Naples, Italy; 5 Clinical and Translational Research Unit, Santobono-Pausilipon Children’s Hospital, Naples, Italy

**Keywords:** colonoscopy, constipation, mucosal ulceration, rectal bleeding, stercoral colitis

## Abstract

A 10-year-old patient was admitted to the pediatric sub-intensive care unit due to massive rectal bleeding and severe acute anemia. His medical background included Asperger syndrome and a history of chronic constipation not adequately addressed by medical therapy. Colonoscopy showed two deep ulcers in the rectum and sigmoid colon of approximately 4 cm in diameter, which were diagnosed as stercoral ulcers related to stercoral colitis, a rare inflammatory form of colitis that occurs in patients with refractory constipation. Stercoral colitis has been mainly described in elderly patients with dementia or in bedbound patients and only occasionally in adults with psychiatric disorders. To the best of our knowledge, this is the first case of massive rectal bleeding from stercoral ulcers in a pediatric patient.

## Introduction

A 10-year-old patient was admitted to the pediatric sub-intensive care unit due to massive rectal bleeding and severe acute anemia. His medical background included Asperger syndrome diagnosed at the age of 5 years and a history of chronic constipation, the onset of which dated back to his first months of life. Delay in reaching sphincter control was also reported. His constipation had been characterized by bowel movements of hard stools (grade 1 or 2 on the Bristol scale) once every 1–2 weeks, along with recurrent episodes of soiling.

At admission to our hospital, the child appeared in poor clinical conditions, pale, and severely dehydrated, with prolonged capillary refill and reduced diuresis (heart rate, 140/min; blood pressure, 90/40 mmHg; respiratory rate, 20/min; oxygen saturation, 95%; temperature, 36°C). He had abdominal distension with evident abdominal superficial veins and a palpable mass in the left iliac fossa and hypogastrium. Two episodes of copious rectal bleeding were reported in the last 24 h, with no history of bleeding previously. The last bowel movement of a small amount of hard stool was reported around 15 days before the admission. The maintenance laxatives were stopped 4 months before the admission as the parents were concerned about liquid stools, which were most likely soiling. His recent medical history reported the assumption of a non-steroidal anti-inflammatory drug (NSAID), in particular ibuprofen, at a dosage of 40 mg kg^−1^ day^−1^ for 9 days for an upper respiratory tract infection.

Blood tests revealed metabolic acidosis [pH 7.23, pCO_2_ = 45.4, lactate = 7 mmol/L, HCO_3_^−^ = 10 mmol/L, and blood base excess (BE-B) = −16], severe anemia [red blood cells (RBCs) = 1,656,000/μl, hemoglobin = 4.5 g/dl, hematocrit = 12.3%, and platelets = 636,000/μl], hypoalbuminemia (2.1 g/dl), C-reactive protein of 47.58 mg/L [normal value (n.v.) = 0–5], procalcitonin 0.58 ng/m (n.v. < 0.5), erythrocyte sedimentation rate of 32 mm, and normal clotting.

Abdominal radiographic imaging revealed hyperdistension of the rectum, sigmoid, and descending colon up to 10 cm with a marked fecal impaction.

Abdominal ultrasound showed a fecal mass located in the rectosigmoid region and the left colon, dislocating the bladder. These segments showed anterior wall thickening with a stratified appearance and intermediate hypoechogenicity. The perivisceral adipose tissue was thickened and hyperechoic in the left iliac fossa, where venous ectasia was also observed.

**Figure 1:**
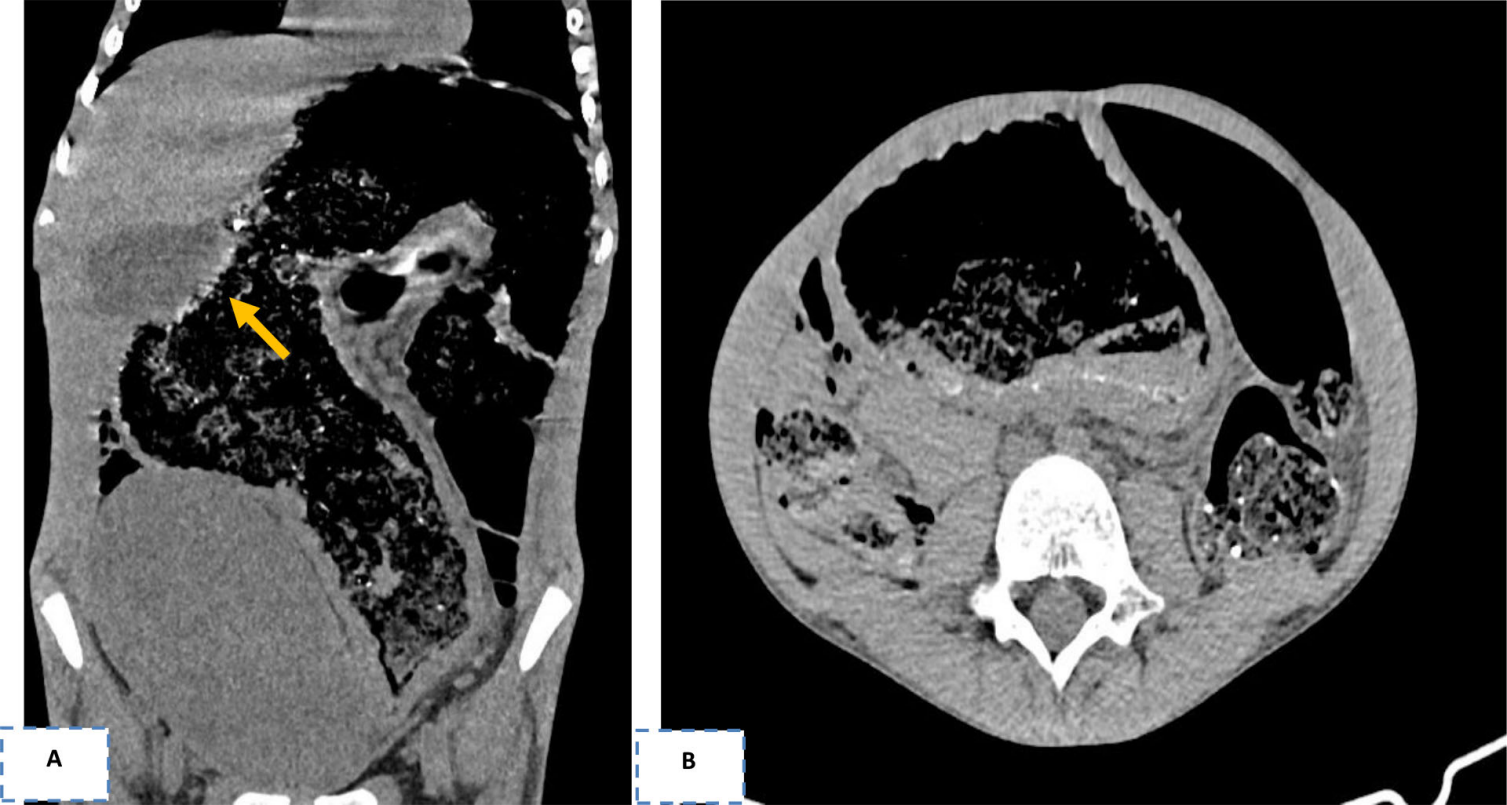
**(A)** Severe colonic distension with fecal impaction from the transverse colon to the rectum (maximum diameter, 11 cm at the level of the overdistended sigmoid). The sigmoid appears dolic and extended from the hepatic flexure to the pelvic excavation, with a tortuous course. Post-contrast enhancement in the arterial phase shows active contrast extravasation at the sigmoid–rectum junction (arrow), consistent with ongoing hemorrhage. **(B)** Concentric wall thickening (maximum, 10 mm) characterized by hyperemia of the mucosal side and edema of the submucosa, which appears hypovascularized both in the descending colon and the sigma. Moreover, the abdominal compartment has an increased anteroposterior diameter in axial view, the abdominal aorta presents a reduction in caliber (maximum, 6.5 mm) compared with the thoracic tract, and the bladder appears deviated cranially and to the right.

Computed tomography angiography (CTA) documented severe colonic distension, fecal impaction from the transverse colon to the rectum, concentric wall thickening, and a marked vascular engorgement of the venous axes of the mesentery. Moreover, post-contrast enhancement in the arterial phase showed active contrast extravasation at the sigmoid–rectum junction, consistent with ongoing hemorrhage (**Figures 1A, B**).

Immediately, a concentrated RBC transfusion of 10 ml/kg was performed twice. Treatment with 2 mg kg^−1^ day^−1^ omeprazole intravenously in two doses and 2 μg kg^−1^ h^−1^ octreotide continuous infusion intravenously was commenced soon after, in the suspicion of possible small bowel bleeding. Due to the persistence of rectal bleeding, the patient underwent esophagogastroduodenoscopy and colonoscopy, which showed a normal upper gastrointestinal tract and was not feasible in the lower tract due to the presence of massive hard stool, which was in part manually removed during the procedure, leading to spontaneous resolution of rectal bleeding.

**Figure 2:**
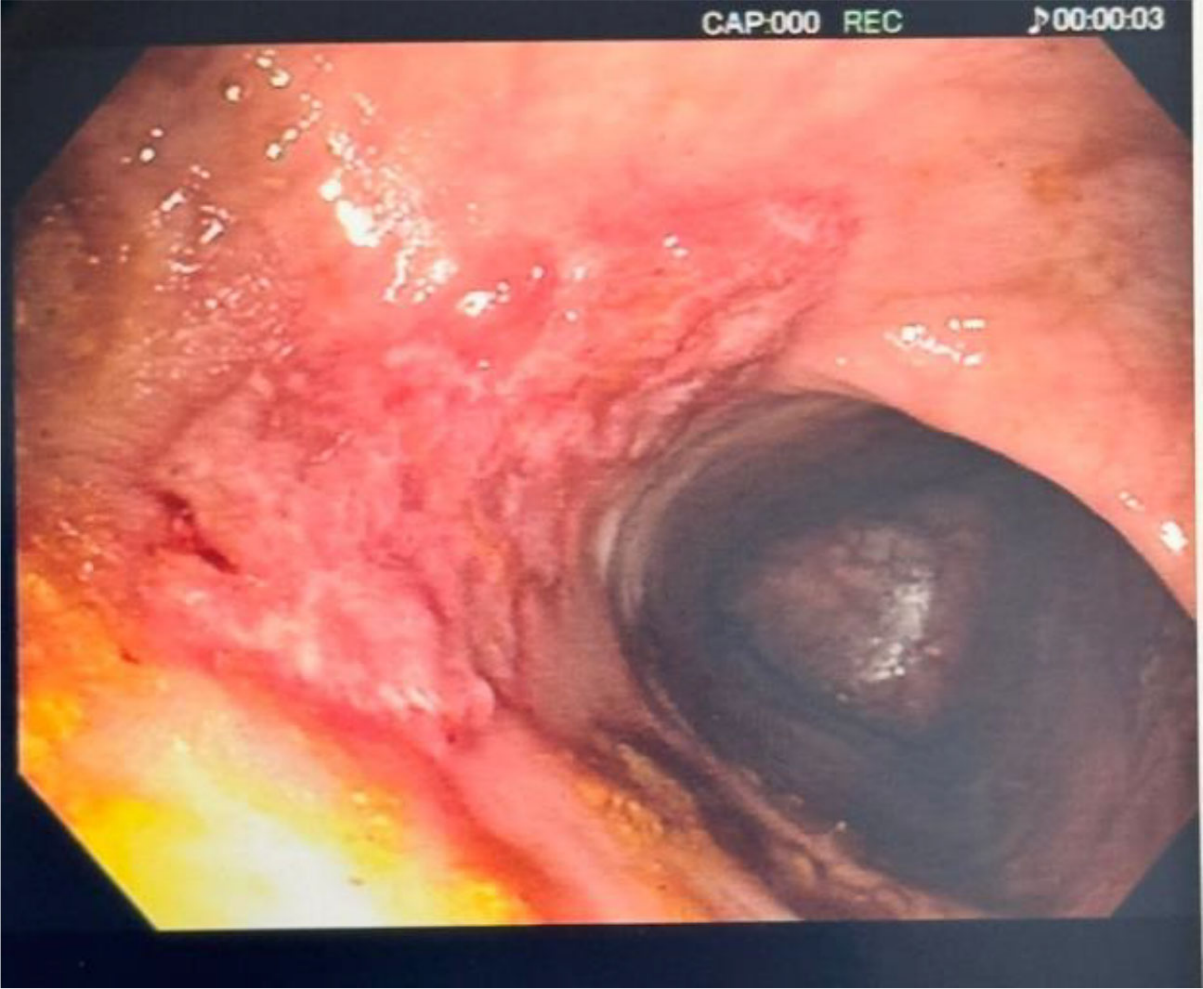
Vast deep stercoral ulcer within the patient’s rectum, covered by fibrin, with elevated edges consistent with stasis ulcer.

In the following days, after bowel cleansing was reached with administration of 7 g kg^−1^ day^−1^ of polyethylene glycol for 5 days, a repeat colonoscopy showed two deep ulcers in the rectum and sigmoid colon of approximately 4 cm in diameter covered by fibrin (**Figure 2**).

Biopsies were performed in the ascending/transverse/descending colon, sigmoid colon, and rectum (these were taken at the level of the ulcers). Microscopic examination of fragments of the ascending/transverse/descending colonic mucosa showed preserved glandular architecture and chronic inflammatory infiltrate, predominantly lymphoplasmacytic, in the lamina propria with follicular-like organization. Microscopic examination of fragments of the sigmoid colon and rectum showed focal areas of erosion, with a chronic–active inflammatory infiltrate in the lamina propria, which, in one of the fragments, was mixed with fibro-histiocytic cells and new vessel formation, consistent with granulation tissue. Histology examinations showed, in the sigmoid colon and rectum, evidence of mucosal erosion with chronic–active inflammatory infiltration and granulation tissue.

After colonoscopy, maintenance treatment for chronic constipation was started with both polyethylene glycol 0.7 g kg^−1^ day^−1^ and bisacodyl 0.4 mg kg^−1^ day^−1^, which led to spontaneous daily passage of soft stool and a reduction of colonic distension, as evidenced by the abdominal ultrasounds performed.

The patient required parenteral nutrition for 15 days. Subsequently, he was initially fed through a nasogastric tube with formulated milk and later transitioned to an oral diet with a free diet.

Diagnostic workup of the causes of chronic constipation included a sweat test, which resulted normal; anorectal manometry, which showed the presence of recto-anal inhibitory reflex; colonic transit study, which showed slow transit constipation; and lumbar spine magnetic resonance imaging, which did not reveal any spine abnormalities.

The patient was discharged after a month of hospitalization on treatment with polyethylene glycol 0.7 mg kg^−1^ day^−1^ and bisacodyl 0.4 mg kg^−1^ day^−1^. At the 10-month follow-up, he had spontaneous daily bowel movements with normal stool consistency. He had not experienced any further episodes of gastrointestinal bleeding. He followed a free diet with good weight gain, and laboratory tests showed normal inflammatory markers.

Stercoral colitis (SC) is a rare inflammatory form of colitis that occurs in patients with refractory constipation, accounting for less than 1%–2% of colonic perforations. Clinical presentation is often insidious, with vague abdominal pain and constipation, but may abruptly progress to perforation and fecal peritonitis. Once complicated, it is associated with high morbidity and mortality, with reported mortality rates of 30%–50%. It has been mainly described in elderly patients with dementia or in bedbound patients and only occasionally in adults with psychiatric disorders. The impacted fecal material leads to increased intraluminal pressure and distension of the colon, resulting in a reduced blood flow, edema, and inflammation that can lead to pressure necrosis, mucosal ulceration with subsequent rectal bleeding, perforation, peritonitis, and sepsis (1). Colon perforation typically occurs in the sigmoid colon and may even represent the first clinical presentation of a stercoral ulcer.

A correlation has been reported between the prolonged use of NSAIDs and nonspecific colitis with ulcers complicated by chronic bleeding (2). Therefore, it is extremely likely that the assumption of ibuprofen over the days prior to admission may have contributed to the clinical presentation of our patient.

To the best of our knowledge, this is the first case of massive rectal bleeding from stercoral ulcers in a pediatric patient. In 2023, a stercoral rectal perforation was described in a pediatric patient affected by chronic constipation; however, no rectal bleeding was reported (3).

As possible comorbidities for SC, our patient presented a neurodevelopmental disorder included within the autism spectrum disorders along with the recent NSAID use, which could have played a role in the development of such severe rectal bleeding. We believe that pediatricians should be aware of this possible, albeit rare, complication of inadequately treated chronic constipation. Close monitoring of bowel habits and the use of laxatives as a maintenance therapy are necessary for patients with chronic constipation, especially in those with potential risk factors for SC.

## Data Availability

The raw data supporting the conclusions of this article will be made available by the authors, without undue reservation.
